# Poly[[bis­[μ-1,2-bis­(4-pyrid­yl)ethene]bis­(trichloro­acetato)­cadmium(II)] monohydrate]

**DOI:** 10.1107/S1600536810049457

**Published:** 2010-12-04

**Authors:** Jin Hoon Kim, Cheal Kim, Youngmee Kim

**Affiliations:** a Department of Fine Chemistry, and Eco-Product and Materials Education Center, Seoul National University of Science and Technology, Seoul 139-743, Republic of Korea; b Department of Chemistry and Nano Science, Ewha Womans University, Seoul 120-750, Republic of Korea

## Abstract

In the crystal structure of the title compound, {[Cd(C_2_Cl_3_O_2_)_2_(C_12_H_10_N_2_)_2_]·H_2_O}_*n*_, the Cd^II^ ion lies on a twofold rotation axis and 1,2-bis­(4-pyrid­yl)ethene ligands bridge symmetry-related Cd^II^ ions, forming a two-dimensional structure. Two trichloro­acetate ligands complete the coordination around the Cd^II ^ion, forming a distorted octa­hedral environment. In the crystal, solvent water mol­ecules, which also lie on twofold rotation axes, form inter­molecular O—H⋯O hydrogen bonds, which connect the two-dimensional structure into a three-dimensional network. The crystal studied was an inversion twin, the refined ratio of twin components being 0.75 (4):0.25 (4).

## Related literature

For background to self-assembly processes, see: Batten & Robson (1998 ▶); Moler *et al.* (2001 ▶); Moulton & Zaworotko (2001 ▶); Kim  (2002) ▶; Evans & Lin (2002 ▶). For supra­molecular assemblies, see: Sauvage & Hosseini (1995 ▶); Fujita *et al.* (2001 ▶); Aromí *et al.* (2006 ▶). For optical sensors and heterogeneous catalysts, see: Yoo *et al.* (2003 ▶); Takizawa *et al.* (2003 ▶); Hong *et al.* (2004 ▶); Kitagawa *et al.* (2004 ▶); Hong *et al.* (2005 ▶); Han *et al.* (2006 ▶). 
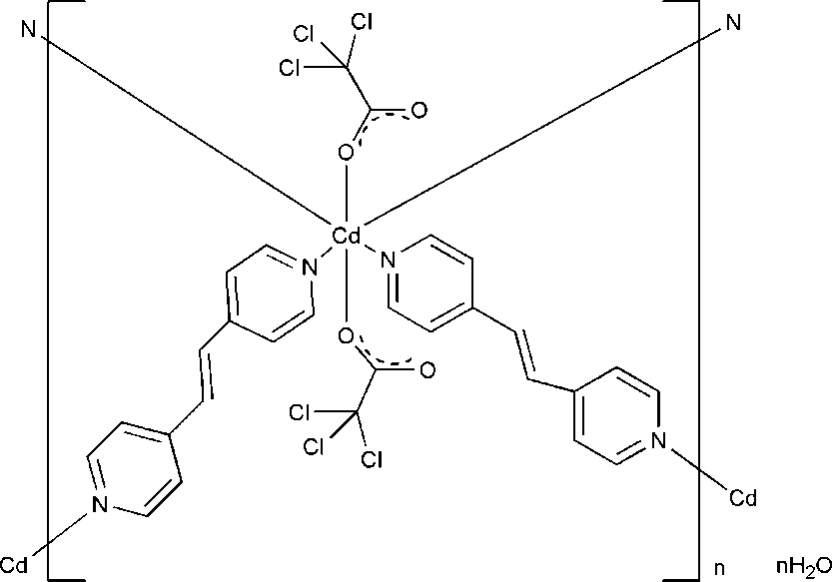


## Experimental

### 

#### Crystal data

[Cd(C_2_Cl_3_O_2_)_2_(C_12_H_10_N_2_)_2_]·H_2_O
*M* *_r_* = 819.60
Orthorhombic, 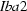

*a* = 19.618 (4) Å
*b* = 9.5760 (19) Å
*c* = 17.517 (4) Å
*V* = 3290.8 (11) Å^3^
*Z* = 4
Mo *K*α radiation
μ = 1.19 mm^−1^
*T* = 293 K
0.25 × 0.20 × 0.20 mm

#### Data collection

Bruker SMART CCD area-detector diffractometer
Absorption correction: multi-scan (*SADABS*; Sheldrick, 1996 ▶) *T*_min_ = 0.751, *T*_max_ = 0.788
8746 measured reflections
3146 independent reflections
2738 reflections with *I* > 2σ(*I*)
*R*_int_ = 0.025

#### Refinement

*R*[*F* ^2^ > 2σ(*F* ^2^)] = 0.032
*wR*(*F* ^2^) = 0.085
*S* = 1.07
3146 reflections
205 parameters
1 restraint
H atoms treated by a mixture of independent and constrained refinement
Δρ_max_ = 0.69 e Å^−3^
Δρ_min_ = −0.49 e Å^−3^
Absolute structure: Flack (1983 ▶), 1477 Friedel pairs
Flack parameter: 0.25 (4)

### 

Data collection: *SMART* (Bruker, 1997 ▶); cell refinement: *SAINT* (Bruker, 1997 ▶); data reduction: *SAINT*; program(s) used to solve structure: *SHELXS97* (Sheldrick, 2008 ▶); program(s) used to refine structure: *SHELXL97* (Sheldrick, 2008 ▶); molecular graphics: *SHELXTL* (Sheldrick, 2008 ▶); software used to prepare material for publication: *SHELXTL*.

## Supplementary Material

Crystal structure: contains datablocks I, global. DOI: 10.1107/S1600536810049457/lh5170sup1.cif

Structure factors: contains datablocks I. DOI: 10.1107/S1600536810049457/lh5170Isup2.hkl

Additional supplementary materials:  crystallographic information; 3D view; checkCIF report

## Figures and Tables

**Table 1:** Hydrogen-bond geometry (Å, °)

*D*—H⋯*A*	*D*—H	H⋯*A*	*D*⋯*A*	*D*—H⋯*A*
O1*S*—H1*S*⋯O3^i^	0.97 (6)	1.97 (6)	2.924 (6)	167 (5)

Symmetry code: (i) 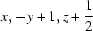.

**Fig. 1.**
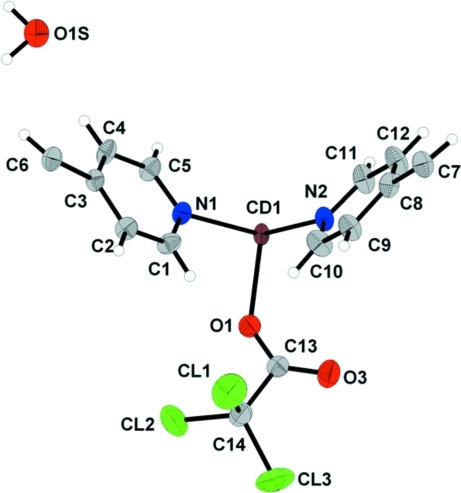
The asymmetric unit of the title compound with labeling scheme. Displacement ellipsoids are shown at the 50% probability level. The water molecule in the asymmetric unit is half occupancy.

**Fig. 2.**
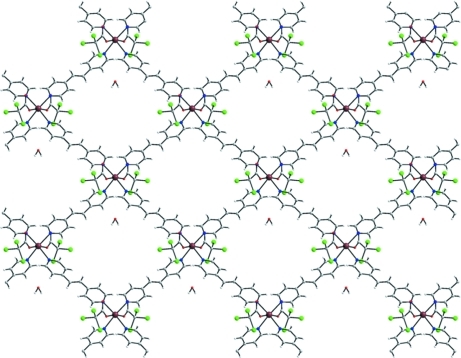
Part of a two-dimensional sheet of the title compound.

## supplementary crystallographic information

### Comment

Self-assembly processes involving metal ions and organic ligands directed by either metal coordination or hydrogen bonds have attracted much attention in the field of supramolecular chemistry and current coordination chemistry (Batten, *et al.*,1998; Moler,*et al.*, 2001; Moulton, *et al*., 2001; Kim, *et al.*, 2002; Evans, *et al.*, 2002). The use of rigid or flexible spacer ligands is of considerable interests in recent years owing to their potential as building blocks for supramolecular assemblies (Sauvage, *et al.*,1995; Fujita, *et al.*, 2001; Aromí, *et al.*, 2006) and their ability to act as optical sensors and heterogeneous catalysts (Yoo, *et al.*, 2003; Takizawa, *et al.*, 2003; Hong, *et al.*, 2004; Kitagawa, *et al.*, 2004; Hong, *et al.*, 2005; Han, *et al.*, 2006). In our attempt to investigate the design and control of the self-assembly of coordination polymers with the rigid bridging ligands, we have employed Cd^II^ to develop a new polymeric complex with the ligand 1,2-bis(4-pyridyl)ethene. We report herein the crystal structure of the title compound [Cd(O_2_C_2_Cl_3_)_2_(C_12_H_10_N_2_)_2_]_n _(C_12_H_10_N_2_= 1,2-bis(4-pyridyl)ethene).

The asymmetric unit of the title compound is shown in Fig. 1. The unique Cd^II^ ion lies on a twofold roation axis and 1,2-bis(4-pyridyl)ethene ligands bridge symmetry related Cd^II^ ions to form a two-dimensional structure (Fig. 2). Two trichloroacetato ligands complete the coordination around the Cd^II ^ion to form a distorted octahedral environment. In the crystal structure solvent water molecules, which also lie on twofold roation axes, form intermolecular O-H···O hydrogen bonds which connect the two-dimensional structure into a three-dimensional network.

### Experimental

39.3 mg (0.125 mmol) of Cd(NO_3_)_2_^.^4H_2_O and 41.3 mg (0.25 mmol) of CCl_3_COOH and 15.8 mg (0.125 mmol) of NH_4_OH were dissolved in 4 ml water and carefully layered by 4 ml ethanol of 1,2-bis(4-pyridyl)ethene ligand (47.0 mg, 0.25 mmol). Suitable crystals of the title compound for X-ray analysis were obtained in a month.

### Refinement

H atoms were placed incalculated positions with C—H distances of 0.93 Å (phenyl). They were included in the refinement in riding-motion approximation with Uĩso~(H) = 1.2U~eq~(C). The unique H atom bonded to the water molecule was refined independently with an isotropic displacement parameter.

### Crystal data

**Table:**

[Cd(C_2_Cl_3_O_2_)_2_(C_12_H_10_N_2_)_2_]·H_2_O	*F*(000) = 1632
*M**_r_* = 819.60	*D*_x_ = 1.654 Mg m^−^^3^
Orthorhombic, *I**b**a*2	Mo *K*α radiation, λ = 0.71073 Å
Hall symbol: I 2 -2c	Cell parameters from 3648 reflections
*a* = 19.618 (4) Å	θ = 2.4–26.3°
*b* = 9.5760 (19) Å	µ = 1.19 mm^−^^1^
*c* = 17.517 (4) Å	*T* = 293 K
*V* = 3290.8 (11) Å^3^	Block, colorless
*Z* = 4	0.25 × 0.20 × 0.20 mm

### Data collection

**Table:**

Bruker SMART CCD area-detector diffractometer	3146 independent reflections
Radiation source: fine-focus sealed tube	2738 reflections with *I* > 2σ(*I*)
graphite	*R*_int_ = 0.025
φ and ω scans	θ_max_ = 26.0°, θ_min_ = 2.1°
Absorption correction: multi-scan (*SADABS*; Sheldrick, 1996)	*h* = −23→24
*T*_min_ = 0.751, *T*_max_ = 0.788	*k* = −11→8
8746 measured reflections	*l* = −21→21

### Refinement

**Table:**

Refinement on *F*^2^	Secondary atom site location: difference Fourier map
Least-squares matrix: full	Hydrogen site location: inferred from neighbouring sites
*R*[*F*^2^ > 2σ(*F*^2^)] = 0.032	H atoms treated by a mixture of independent and constrained refinement
*wR*(*F*^2^) = 0.085	*w* = 1/[σ^2^(*F*_o_^2^) + (0.0561*P*)^2^ + 0.189*P*] where *P* = (*F*_o_^2^ + 2*F*_c_^2^)/3
*S* = 1.07	(Δ/σ)_max_ < 0.001
3146 reflections	Δρ_max_ = 0.69 e Å^−^^3^
205 parameters	Δρ_min_ = −0.49 e Å^−^^3^
1 restraint	Absolute structure: Flack (1983), 1477 Friedel pairs
Primary atom site location: structure-invariant direct methods	Flack parameter: 0.25 (4)

### Special details

**Table:**

Geometry. All e.s.d.'s (except the e.s.d. in the dihedral angle between two l.s. planes) are estimated using the full covariance matrix. The cell e.s.d.'s are taken into account individually in the estimation of e.s.d.'s in distances, angles and torsion angles; correlations between e.s.d.'s in cell parameters are only used when they are defined by crystal symmetry. An approximate (isotropic) treatment of cell e.s.d.'s is used for estimating e.s.d.'s involving l.s. planes.
Refinement. Refinement of *F*^2^ against ALL reflections. The weighted *R*-factor *wR* and goodness of fit *S* are based on *F*^2^, conventional *R*-factors *R* are based on *F*, with *F* set to zero for negative *F*^2^. The threshold expression of *F*^2^ > σ(*F*^2^) is used only for calculating *R*-factors(gt) *etc*. and is not relevant to the choice of reflections for refinement. *R*-factors based on *F*^2^ are statistically about twice as large as those based on *F*, and *R*- factors based on ALL data will be even larger.

### Fractional atomic coordinates and isotropic or equivalent isotropic displacement parameters (Å^2^)

**Table:**

	*x*	*y*	*z*	*U*_iso_*/*U*_eq_	
C1	0.1330 (3)	0.5206 (5)	0.3412 (4)	0.0483 (15)	
H1	0.1462	0.4440	0.3121	0.058*	
C2	0.1778 (2)	0.5728 (5)	0.3947 (3)	0.0452 (10)	
H2	0.2203	0.5317	0.4013	0.054*	
C3	0.15908 (19)	0.6871 (4)	0.4386 (2)	0.0370 (9)	
C4	0.0939 (2)	0.7358 (6)	0.4289 (2)	0.0483 (11)	
H4	0.0779	0.8075	0.4598	0.058*	
C5	0.0521 (2)	0.6800 (5)	0.3739 (2)	0.0474 (11)	
H5	0.0086	0.7171	0.3678	0.057*	
C6	0.20672 (19)	0.7556 (5)	0.4922 (2)	0.0454 (12)	
H6	0.1884	0.8097	0.5312	0.055*	
C7	0.2262 (2)	0.7553 (5)	−0.0119 (3)	0.0496 (12)	
H7	0.2086	0.8121	−0.0503	0.060*	
C8	0.17672 (19)	0.6860 (5)	0.0386 (2)	0.0437 (10)	
C9	0.1933 (2)	0.5842 (5)	0.0904 (2)	0.0509 (12)	
H9	0.2381	0.5534	0.0942	0.061*	
C10	0.1447 (3)	0.5281 (7)	0.1361 (4)	0.0547 (16)	
H10	0.1578	0.4583	0.1700	0.066*	
C11	0.0618 (2)	0.6619 (6)	0.0834 (2)	0.0530 (13)	
H11	0.0163	0.6882	0.0794	0.064*	
C12	0.1090 (2)	0.7238 (7)	0.0345 (3)	0.0576 (14)	
H12	0.0949	0.7904	−0.0007	0.069*	
C13	0.0825 (2)	0.1977 (4)	0.1993 (3)	0.0433 (9)	
C14	0.1275 (2)	0.0920 (5)	0.2461 (3)	0.0567 (12)	
Cd1	0.0000	0.5000	0.22812 (5)	0.03111 (11)	
Cl1	0.20978 (7)	0.1774 (2)	0.25364 (11)	0.1014 (6)	
Cl2	0.09814 (9)	0.0658 (2)	0.33949 (9)	0.0954 (5)	
Cl3	0.14021 (12)	−0.06656 (19)	0.19892 (14)	0.1285 (9)	
N1	0.07119 (15)	0.5758 (4)	0.32947 (19)	0.0398 (8)	
N2	0.07875 (16)	0.5669 (5)	0.13543 (19)	0.0403 (8)	
O1	0.05695 (13)	0.2898 (3)	0.23965 (17)	0.0488 (7)	
O3	0.08109 (18)	0.1802 (4)	0.13081 (19)	0.0680 (9)	
O1S	0.0000	1.0000	0.5313 (4)	0.0764 (17)	
H1S	0.021 (3)	0.941 (6)	0.570 (3)	0.059 (14)*	

### Atomic displacement parameters (Å^2^)

**Table:**

	*U*^11^	*U*^22^	*U*^33^	*U*^12^	*U*^13^	*U*^23^
C1	0.044 (3)	0.044 (3)	0.057 (3)	−0.0035 (19)	−0.009 (3)	−0.013 (2)
C2	0.0299 (19)	0.054 (3)	0.052 (2)	0.0011 (19)	−0.0088 (17)	−0.005 (2)
C3	0.0309 (19)	0.045 (2)	0.0349 (19)	−0.0118 (17)	−0.0088 (15)	−0.0007 (16)
C4	0.042 (2)	0.063 (3)	0.040 (2)	−0.001 (2)	−0.0068 (17)	−0.018 (2)
C5	0.034 (2)	0.060 (3)	0.049 (2)	0.005 (2)	−0.0116 (18)	−0.014 (2)
C6	0.039 (2)	0.056 (2)	0.042 (3)	−0.0081 (18)	−0.0075 (17)	−0.0070 (18)
C7	0.0405 (18)	0.065 (3)	0.044 (3)	−0.0112 (19)	0.010 (2)	0.012 (2)
C8	0.0314 (19)	0.061 (3)	0.039 (2)	−0.0055 (18)	0.0066 (17)	−0.002 (2)
C9	0.033 (2)	0.069 (3)	0.050 (3)	−0.001 (2)	0.0095 (18)	0.017 (2)
C10	0.034 (3)	0.069 (3)	0.062 (4)	−0.004 (2)	0.014 (3)	0.018 (3)
C11	0.029 (2)	0.085 (4)	0.045 (2)	−0.003 (2)	0.0042 (17)	0.015 (2)
C12	0.039 (2)	0.083 (4)	0.051 (3)	−0.003 (2)	0.008 (2)	0.026 (3)
C13	0.040 (2)	0.042 (2)	0.048 (2)	−0.0078 (17)	0.0007 (17)	−0.0015 (17)
C14	0.058 (2)	0.059 (3)	0.054 (3)	0.009 (2)	−0.007 (2)	−0.010 (2)
Cd1	0.02345 (16)	0.04508 (18)	0.02480 (16)	−0.01014 (13)	0.000	0.000
Cl1	0.0462 (6)	0.1336 (14)	0.1242 (14)	0.0178 (7)	−0.0158 (8)	−0.0198 (11)
Cl2	0.1164 (13)	0.0992 (12)	0.0706 (9)	0.0192 (10)	0.0012 (9)	0.0275 (9)
Cl3	0.173 (2)	0.0646 (10)	0.148 (2)	0.0378 (12)	−0.0147 (15)	−0.0328 (11)
N1	0.0336 (18)	0.049 (2)	0.0363 (19)	−0.0101 (16)	−0.0033 (14)	−0.0055 (17)
N2	0.0304 (18)	0.057 (2)	0.0334 (18)	−0.0074 (17)	0.0056 (13)	0.0067 (17)
O1	0.0489 (14)	0.0503 (15)	0.0471 (18)	0.0042 (12)	0.0031 (13)	−0.0007 (14)
O3	0.074 (2)	0.078 (2)	0.051 (2)	0.0024 (18)	−0.0073 (16)	−0.0095 (17)
O1S	0.091 (5)	0.078 (4)	0.060 (4)	0.001 (3)	0.000	0.000

### Geometric parameters (Å, °)

**Table:**

C1—N1	1.338 (7)	C10—N2	1.347 (7)
C1—C2	1.379 (8)	C10—H10	0.9300
C1—H1	0.9300	C11—N2	1.330 (6)
C2—C3	1.387 (6)	C11—C12	1.393 (6)
C2—H2	0.9300	C11—H11	0.9300
C3—C4	1.371 (6)	C12—H12	0.9300
C3—C6	1.479 (5)	C13—O3	1.212 (5)
C4—C5	1.374 (6)	C13—O1	1.236 (5)
C4—H4	0.9300	C13—C14	1.573 (6)
C5—N1	1.320 (6)	C14—Cl3	1.746 (5)
C5—H5	0.9300	C14—Cl2	1.753 (5)
C6—C7^i^	1.323 (6)	C14—Cl1	1.815 (5)
C6—H6	0.9300	Cd1—O1^iii^	2.311 (3)
C7—C6^ii^	1.322 (6)	Cd1—O1	2.311 (3)
C7—C8	1.471 (6)	Cd1—N2	2.331 (3)
C7—H7	0.9300	Cd1—N2^iii^	2.331 (3)
C8—C9	1.371 (6)	Cd1—N1	2.373 (3)
C8—C12	1.379 (6)	Cd1—N1^iii^	2.373 (3)
C9—C10	1.356 (7)	O1S—H1S	0.97 (6)
C9—H9	0.9300		
			
N1—C1—C2	122.6 (5)	C8—C12—H12	120.1
N1—C1—H1	118.7	C11—C12—H12	120.1
C2—C1—H1	118.7	O3—C13—O1	131.0 (4)
C1—C2—C3	119.6 (4)	O3—C13—C14	116.0 (4)
C1—C2—H2	120.2	O1—C13—C14	112.9 (4)
C3—C2—H2	120.2	C13—C14—Cl3	113.2 (3)
C4—C3—C2	116.5 (4)	C13—C14—Cl2	113.2 (3)
C4—C3—C6	121.1 (4)	Cl3—C14—Cl2	111.4 (3)
C2—C3—C6	122.3 (4)	C13—C14—Cl1	104.3 (3)
C3—C4—C5	120.8 (4)	Cl3—C14—Cl1	107.4 (3)
C3—C4—H4	119.6	Cl2—C14—Cl1	106.8 (3)
C5—C4—H4	119.6	O1^iii^—Cd1—O1	169.98 (15)
N1—C5—C4	122.5 (4)	O1^iii^—Cd1—N2	98.16 (13)
N1—C5—H5	118.8	O1—Cd1—N2	88.84 (13)
C4—C5—H5	118.8	O1^iii^—Cd1—N2^iii^	88.85 (13)
C7^i^—C6—C3	124.0 (4)	O1—Cd1—N2^iii^	98.16 (13)
C7^i^—C6—H6	118.0	N2—Cd1—N2^iii^	91.69 (17)
C3—C6—H6	118.0	O1^iii^—Cd1—N1	87.28 (12)
C6^ii^—C7—C8	126.0 (4)	O1—Cd1—N1	85.22 (12)
C6^ii^—C7—H7	117.0	N2—Cd1—N1	92.69 (10)
C8—C7—H7	117.0	N2^iii^—Cd1—N1	174.52 (14)
C9—C8—C12	116.7 (4)	O1^iii^—Cd1—N1^iii^	85.22 (12)
C9—C8—C7	124.2 (4)	O1—Cd1—N1^iii^	87.29 (12)
C12—C8—C7	119.1 (4)	N2—Cd1—N1^iii^	174.52 (14)
C10—C9—C8	120.4 (4)	N2^iii^—Cd1—N1^iii^	92.69 (10)
C10—C9—H9	119.8	N1—Cd1—N1^iii^	83.13 (16)
C8—C9—H9	119.8	C5—N1—C1	117.7 (4)
N2—C10—C9	124.1 (5)	C5—N1—Cd1	120.3 (3)
N2—C10—H10	117.9	C1—N1—Cd1	121.8 (3)
C9—C10—H10	117.9	C11—N2—C10	115.8 (4)
N2—C11—C12	123.1 (4)	C11—N2—Cd1	120.0 (3)
N2—C11—H11	118.5	C10—N2—Cd1	123.7 (3)
C12—C11—H11	118.5	C13—O1—Cd1	140.1 (3)
C8—C12—C11	119.8 (5)		
			
N1—C1—C2—C3	0.0 (8)	O1—Cd1—N1—C5	−156.1 (3)
C1—C2—C3—C4	4.1 (6)	N2—Cd1—N1—C5	115.2 (4)
C1—C2—C3—C6	−174.7 (5)	N1^iii^—Cd1—N1—C5	−68.3 (3)
C2—C3—C4—C5	−5.0 (7)	O1^iii^—Cd1—N1—C1	−159.2 (4)
C6—C3—C4—C5	173.9 (4)	O1—Cd1—N1—C1	27.4 (4)
C3—C4—C5—N1	1.8 (8)	N2—Cd1—N1—C1	−61.2 (4)
C4—C3—C6—C7^i^	−158.2 (5)	N1^iii^—Cd1—N1—C1	115.3 (4)
C2—C3—C6—C7^i^	20.6 (7)	C12—C11—N2—C10	−3.0 (8)
C6^ii^—C7—C8—C9	−9.8 (8)	C12—C11—N2—Cd1	169.0 (4)
C6^ii^—C7—C8—C12	170.4 (5)	C9—C10—N2—C11	3.2 (9)
C12—C8—C9—C10	−1.8 (8)	C9—C10—N2—Cd1	−168.5 (5)
C7—C8—C9—C10	178.4 (5)	O1^iii^—Cd1—N2—C11	−31.9 (4)
C8—C9—C10—N2	−0.8 (10)	O1—Cd1—N2—C11	155.3 (4)
C9—C8—C12—C11	1.9 (8)	N2^iii^—Cd1—N2—C11	57.1 (3)
C7—C8—C12—C11	−178.3 (5)	N1—Cd1—N2—C11	−119.6 (4)
N2—C11—C12—C8	0.5 (9)	O1^iii^—Cd1—N2—C10	139.4 (4)
O3—C13—C14—Cl3	−23.4 (5)	O1—Cd1—N2—C10	−33.4 (4)
O1—C13—C14—Cl3	159.9 (3)	N2^iii^—Cd1—N2—C10	−131.5 (5)
O3—C13—C14—Cl2	−151.3 (4)	N1—Cd1—N2—C10	51.8 (4)
O1—C13—C14—Cl2	32.1 (4)	O3—C13—O1—Cd1	−7.5 (7)
O3—C13—C14—Cl1	93.0 (4)	C14—C13—O1—Cd1	168.5 (3)
O1—C13—C14—Cl1	−83.6 (4)	O1^iii^—Cd1—O1—C13	−179.3 (4)
C4—C5—N1—C1	2.5 (7)	N2—Cd1—O1—C13	−44.8 (4)
C4—C5—N1—Cd1	−174.1 (4)	N2^iii^—Cd1—O1—C13	46.8 (4)
C2—C1—N1—C5	−3.3 (7)	N1—Cd1—O1—C13	−137.6 (4)
C2—C1—N1—Cd1	173.2 (4)	N1^iii^—Cd1—O1—C13	139.1 (4)
O1^iii^—Cd1—N1—C5	17.2 (3)		

Symmetry codes: (i) −*x*+1/2, −*y*+3/2, *z*+1/2; (ii) −*x*+1/2, −*y*+3/2, *z*−1/2; (iii) −*x*, −*y*+1, *z*.

### Hydrogen-bond geometry (Å, °)

**Table:**

*D*—H···*A*	*D*—H	H···*A*	*D*···*A*	*D*—H···*A*
O1S—H1S···O3^iv^	0.97 (6)	1.97 (6)	2.924 (6)	167 (5)

Symmetry codes: (iv) *x*, −*y*+1, *z*+1/2.
